# Channel Exchanging for RGB-T Tracking

**DOI:** 10.3390/s21175800

**Published:** 2021-08-28

**Authors:** Long Zhao, Meng Zhu, Honge Ren, Lingjixuan Xue

**Affiliations:** 1 College of Information and Computer Engineering, Northeast Forestry University, Harbin 150040, China; zhaolong@nefu.edu.cn (L.Z.); zhumeng913@nefu.edu.cn (M.Z.); xuelingjixuan@nefu.edu.cn (L.X.); 2 Big Data Institute, East University of Heilongjiang, Harbin 150066, China; 3 Forestry Intelligent Equipment Engineering Research Center, Harbin 150040, China

**Keywords:** channel exchanging, RGB-T object tracking methods, dual-modal data

## Abstract

It is difficult to achieve all-weather visual object tracking in an open environment only utilizing single modality data input. Due to the complementarity of RGB and thermal infrared (TIR) data in various complex environments, a more robust object tracking framework can be obtained using video data of these two modalities. The fusion methods of RGB and TIR data are the core elements to determine the performance of the RGB-T object tracking method, and the existing RGB-T trackers have not solved this problem well. In order to solve the current low utilization of information intra single modality in aggregation-based methods and between two modalities in alignment-based methods, we used DiMP as the baseline tracker to design an RGB-T object tracking framework channel exchanging DiMP (CEDiMP) based on channel exchanging. CEDiMP achieves dynamic channel exchanging between sub-networks of different modes hardly adding any parameters during the feature fusion process. The expression ability of the deep features generated by our data fusion method based on channel exchanging is stronger. At the same time, in order to solve the poor generalization ability of the existing RGB-T object tracking methods and the poor ability in the long-term object tracking, more training of CEDiMP on the synthetic dataset LaSOT-RGBT is added. A large number of experiments demonstrate the effectiveness of the proposed model. CEDiMP achieves the best performance on two RGB-T object tracking benchmark datasets, GTOT and RGBT234, and performs outstandingly in the generalization testing.

## 1. Introduction

**Figure 1:**
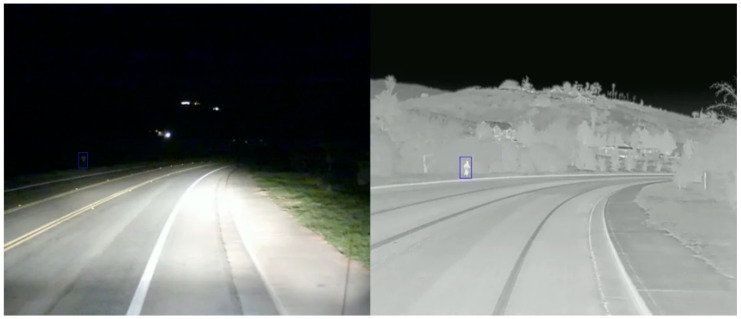
RGB image (**left**) and TIR image (**right**) under low illumination.

**Figure 2:**
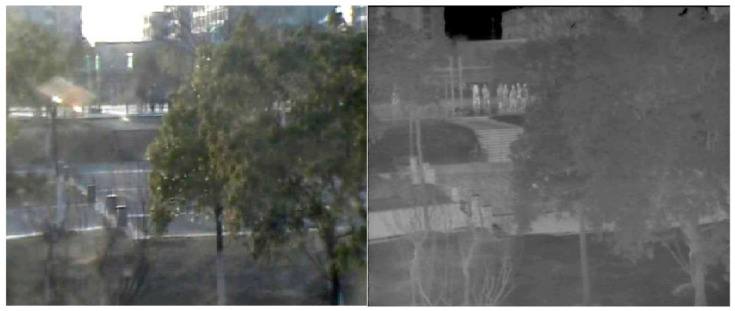
RGB image (**left**) and TIR image (**right**) under strong light conditions.

Although the object tracking method based on visible images has made much breakthrough in solving target state transition and similar objects interference in recent years, the performance of the tracker under specific environments decreases significantly, such as low illumination, strong light, rain, haze, etc. The main reason is that the quality of RGB images produced by the visible light camera is extremely poor [1] in the above environment. However, a thermal infrared camera can produce high-quality TIR images in the above environment. Thermal infrared cameras are not sensitive to light conditions and have a strong penetrating ability. They can capture infrared radiation of 0.75–13 μm wavelength from objects above absolute zero temperature and form the single-channel grayscale images of better quality [2]. We can clearly see the outline of people from the TIR image in Figure 1 (right), while the outline of people in the RGB image (left) is extremely fuzzy. We can clearly know the number of people from the TIR image in Figure 2 (right), while the number of people in the RGB image (left) cannot be seen clearly at all.

**Figure 3:**
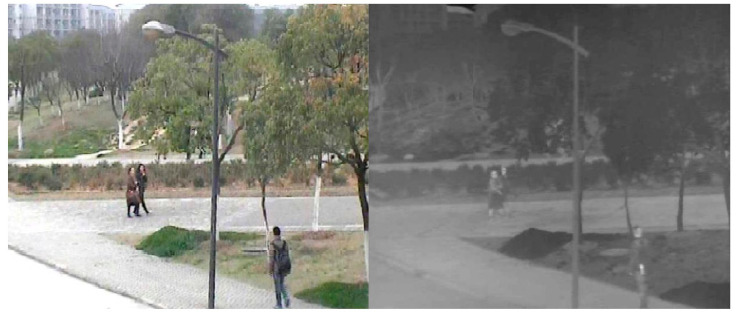
RGB image (**left**) and TIR image (**right**) when the trajectories of two people overlap.

Although thermal infrared cameras can generate images of higher quality than RGB images in specific environments, such as low illumination, strong light, rain, haze, etc., TIR images have low resolution and would easily lose information, such as colors, target object edges, and geometric textures. Thermal infrared cameras are sensitive to temperature, and the effect of thermal infrared images is very poor especially when objects with similar temperatures overlap, as shown in Figure 3. In the RGB image (left) in Figure 3, we can clearly see that a tall woman in dark clothes is partially obscured by a short woman in light clothes. However, in the TIR image (right), due to hot cross, we cannot recognize this tall woman.

### 1.1. Shortcomings of Existing RGB-T Trackers

With the lower cost of multi-spectral sensors, it has become easy to equip the system with a dual-mode camera system including both thermal infrared and visible sensors. Thus, researchers naturally think of designing an object tracking method fusing RGB and TIR data, which is more beneficial to complete all-weather object tracking in an open environment. The core factors that determine the performance of the RGB-T tracking method are whether the robust RGB and TIR features can be extracted and how to effectively utilize the feature data of these two modalities. Currently, the data fusion methods of the excellent RGB-T tracking methods on RGBT234 [2] and GTOT [3] almost adopt the depth feature aggregation or alignment of the two modalities of RGB and TIR. Convergence based fusion tends to underestimate intra-modal propagation. Alignment based fusion maintains intra-modal propagation, but because it only utilizes training alignment loss to exchange weak messages, it has always been unable to achieve effective inter-modal fusion [4].

At present, the most common benchmark datasets, GTOT and RGBT234, for evaluating the performance of RGB-T tracking methods have not many video sequences, insufficient scene types, and generally short video sequences. Such benchmark datasets are not enough to accurately measure the true performance of the tracker. For example, mfDiMP [5], the champion in the VOT-RGBT2019 challenge, only ranks third in EAO on the public dataset, but is the best in EAO in the sequestered dataset, as shown in Table 1.

From Table 1, we can know that the performance of even the best RGB-T tracking framework has dropped significantly in the sequestered dataset. The generalization ability of mfDiMP is relatively good, and this is the reason for its first rank. mfDiMP is not trained on the common benchmark datasets GTOT and RGBT234 (the 60 video sequences of the VOT-RGBT2019 public dataset are all selected from RGBT234) for RGB-T object tracking like other frameworks, while it is trained on artificially synthetic dataset GOT10K-RGBT. mfDiMP utilizes the RGB modality images in the GOT10K [10] dataset to generate the aligned TIR images by using the image-to-image translation method. mfDiMP finally uses synthetic dataset GOT10K-RGBT containing RGB and TIR dual-modality video sequences to complete neural network training. Additionally, mfDiMP is a tracking framework based on DiMP [11], which inherits the powerful target and background discrimination capabilities of DiMP. mfDiMP can quickly capture the changes in the target and background than the other four tracking frameworks, and it is also more suitable to track targets that have not been seen during the training process. We find that mfDiMP directly concatenates the depth features of RGB mode and TIR mode, and then uses 1 × 1 convolution to perform a dimensionality reduction operation. Finally, the fused features are input into the IoU predictor and model predictor. The hyperparameter-based feature aggregation method it utilizes would reduce the model’s representation ability in unique characteristic of the original modality, and this feature fusion method limits the improvement of its performance.

### 1.2. Our Innovation

Inspired by [4], we propose an RGB-T object tracking framework CEDiMP based on channel exchanging. Our proposed method also uses DiMP as the baseline tracker, but we use the channel exchanging method to fuse the data of RGB mode and TIR mode. Channel exchanging is a multi-modal data fusion method with no parameter that can dynamically exchange channels between different modes of sub-networks, which makes our feature representation model possess powerful representation abilities in multi-modal common features and single-modal unique features. We utilize the batch normalization (BN) [12] scale factor (i.e., *γ*) as the importance measurement of each corresponding channel, and replace the channel whose factor is close to zero with the value of another modality. This information exchanging is parameter-free and adaptive, because it is dynamically controlled by the scaling factor determined by the training itself.

In summary, the main contributions of this paper are as follows:

- We propose a novel RGB-T object tracking framework based on channel exchanging. As far as we know, it is the first time that the channel exchanging method has been used to fuse RGB and TIR data for the RGB-T object tracking framework. The data fusion method based on channel exchanging is more efficient than the previous methods.
- In order to improve the generalization performance and long-term tracking ability of the RGB-T tracker, we utilize the trained image translation model for the first time to generate the TIR dataset LaSOT-TIR based on the RGB long-term tracking dataset LaSOT [13]. After training on LaSOT-RGBT, the generalization performance and the ability of long-term tracking have significantly improved.
- Our proposed method not only achieves the best performance on GTOT and RGBT234, but also outperforms existing methods in the evaluation test of sequestered video sequences. Our advantage is especially prominent in the long-term object tracking task.

## 2. Related Work

Initially, researchers only focused on the study of single-modal object tracking methods. With the deepening of research, considering the complementarity of RGB and TIR modalities, tracking algorithms based on the fusion of RGB and TIR data have attracted more and more attention. The cost of multi-spectral sensors has become lower and lower, reducing the threshold of research in this area.

### 2.1. Single-Modal Tracking

**RGB trackers.** The RGB tracker is the most common single-modal tracker. RGB trackers that perform well in accuracy and robustness currently are generally based on appearance modeling. Correlation filtering [14,15,16,17,18,19] is a typical method of learning object appearance model. The above-mentioned correlation filtering methods all solve the tracking problem by learning the appearance model of the target in the first frame. Considering that object tracking can be regarded as a serialized detection problem, the target and background are constantly changing during the tracking process. In order to improve the discriminative ability of the tracker, the latest trackers based on correlation filtering utilize online update to perform the target background classification [20,21,22,23,24]. In addition to using correlation filtering methods, a similarity measure can also be used to locate target objects. This method of using a similarity measure is generally based on the Siamese network [25,26,27,28,29] for end-to-end learning. The Siamese network trackers mentioned above are anchor-based Siamese trackers, except for SiamFC [25]. The anchor-based Siamese tracker has made significant improvements in accuracy, but due to its lack of robustness, further improvements in performance are limited. In order to further improve the robustness of the tracker based on the Siamese network, researchers have proposed the Siamese network tracking framework based on the anchor-free idea [30,31,32] recently. Their accuracy and robustness outperform trackers based on anchor-based ideas on multiple benchmark datasets.

**TIR trackers.** In order to perform robust object tracking under extreme visual conditions, such as darkness, strong light, rain and haze, some researchers have carried out research on object tracking methods based on TIR data. Due to the scarcity of large-scale benchmark datasets for the training and evaluation of TIR object tracking, most TIR object tracking frameworks use manual features. In the VOT-TIR2017 challenge, the top three algorithms [33,34,35] all use manual features. The multi-layer convolutional features for thermal infrared tracking (MCFTS) proposed by Liu Qiao et al. [36] is one of the few methods based on depth features. MCFTS first utilizes a pre-trained convolutional neural network to extract multiple convolutional layer features of thermal infrared objects, and then constructs multiple weak trackers with corresponding convolutional layer features using correlation filters. These weak trackers give a response map of the target location. Finally, MCFTS proposes an integrated method to merge these response maps into a stronger response map. Additionally, MCFTS also proposes a simple and effective scale estimation strategy to improve tracking accuracy. However, the performance of MCFTS is limited by the depth features learned from RGB images for pre-training, and it is less effective in accuracy representing of thermal infrared tracking objects. In order to solve above problems, Liu Qiao et al. have published a paper on AAAI 2020 [37]. This paper has proposed a TIR feature model based on multi-task driven method. This model simultaneously learns the discriminative features and fine-grained correlation features for thermal infrared data. This method has achieved excellent performance on TIR tracking tasks.

### 2.2. Modality Fusion Tracking

Because the fusion of RGB and TIR data more easily achieves all-weather object tracking in the open environment, the researches on RGB-T object tracking methods become more and more popular. From the perspective of data fusion, the RGB-T object tracking framework can be roughly divided into traditional methods [38,39], sparse representation (SR)-based [40,41,42,43,44], graph-based [45,46,47], correlation filter-based [48,49,50,51], and deep learning-based approaches. Earlier studies used manual features to perform the appearance modeling of the target object. These manual features are often invalid when the target scale or lighting conditions change drastically, or the target object move quickly. It is difficult to further improve the robustness of the RGB-T object tracking method using manual features for target representation. Since 2016, the group of Li Chenglong has produced relatively large-scale RGB-T object tracking benchmark datasets GTOT [3], RGBT210 [45], and the latest RGBT234 [2]. With the emergence of these datasets, some excellent RGB-T object tracking methods based on depth features have gradually shown their superior performance [52,53,54,55,56]. The performance improvement of these methods is due to the powerful feature expression ability of deep features. However, these methods are limited by the datasets used for neural network training [2,3,45], which have problems including insufficient scale, generally short video sequences, insufficient scenes, and generally poor generalization performance. Table 2 can more intuitively express the similarities and differences of current mainstream RGB-T tracking methods.

## 3. Methods

In order to improve the efficiency of the data fusion of RGB and TIR modalities and further improve the performance of the RGB-T tracker, we propose the RGB-T object tracking framework CEDiMP based on channel exchanging. Both CEDiMP and mfDiMP [5] use DiMP [11] as the baseline tracker, but unlike mfDiMP, CEDiMP does not utilize aggregation in feature fusion. In order to improve the long-term object tracking ability and generalization ability of CEDiMP, we have also trained on our synthetic dataset LaSOT-RGBT, in addition to training on the synthetic dataset GOT10K-RGBT.

### 3.1. RGB and TIR Feature Fusion Based on Channel Exchanging

The well-known single-target tracking framework SiamRPN++ [28] has proved with the experiments that although the depth feature can have 256 channels or more, only a few channels have high response during the tracking process. This indicates that the depth features are often sparse, and the concatenation of features from two modalities by mfDiMP makes the depth features sparser. Sparse features will significantly reduce the feature expression ability of the appearance model. Inspired by [4], we perform channel exchanging of the RGB and TIR modalities. Specifically, we utilize the scale factor of batch normalization (BN) [12] (i.e., γ) to measure the importance of each corresponding channel. If the scale factor corresponding to a specific channel of the current modality is close to zero, then we replace the value of the current channel with the value of the corresponding channel of another modality.

BN layer is widely used in deep learning, which can eliminate covariate drift and improve generalization ability. We define $x_{v,l}$ as the feature maps of the l-th layer of the RGB branch, and $x_{v,l,c}$ represents the c-th channel. $x_{t,l}$ is the feature maps of the l-th layer of the TIR branch, and $x_{t,l,c}$ represents the c-th channel. BN layer normalizes $x_{v,l}$ and $x_{t,l}$, and then performs affine transformation to obtain Equation (1) and Equation (2), respectively:

(1) $${x^{\prime }}_{v,l,c}=\gamma_{v,l,c}\frac{x_{v,l,c}-\mu_{v,l,c}}{\sqrt{\sigma^{2}{}_{v,l,c}+\epsilon }}+\beta_{v,l,c}$$

(2) $${x^{\prime }}_{t,l,c}=\gamma_{t,l,c}\frac{x_{t,l,c}-\mu_{t,l,c}}{\sqrt{\sigma^{2}{}_{t,l,c}+\epsilon }}+\beta_{t,l,c}$$

where $\mu_{v,l,c}$ and $\sigma_{v,l,c}$, respectively, represent all the activated mean and standard deviation of the current mini-batch data of the RGB branch at all pixel positions (H and W). $\mu_{t,l,c}$ and $\sigma_{t,l,c}$, respectively, represent all the activated mean and standard deviation of the current mini-batch data of the TIR branch at all pixel positions (H and W). $\gamma_{v,l,c}$ and $\beta_{v,l,c}$ are the trainable scale factor and offset in the RGB branch, respectively, $\gamma_{t,l,c}$ and $\beta_{t,l,c}$ are the trainable scale factor and offset in the TIR branch; ϵ is a small constant that can avoid the division by zero. The input of the (l+1)-th layer of RGB and TIR is ${x^{\prime }}_{v,l,c}$ and ${x^{\prime }}_{t,l,c}$, respectively, which is the output of the l-th layer.

In Equations (1) and (2), $\gamma_{v,l,c}$ and $\gamma_{t,l,c}$ evaluate the correlation between the input and output of the l-th layer during the training process of the RGB and TIR branches, respectively. If $\gamma_{v,l,c}$ approaches 0, the gradient of the loss rate of $x_{v,l,c}$ will also approach 0. The relationship between $x_{t,l,c}$ and $\gamma_{t,l,c}$ is the same. Whether it is $x_{v,l,c}$ or $x_{t,l,c}$, as long as the value approaches 0, it will lose its effect in the final prediction. In other words, channel c becomes a redundant channel. To this end, we set a threshold θ. If $\gamma_{v,l,c}<\theta$ and $\gamma_{t,l,c}>\theta$, the feature of channel c in the TIR branch is used to replace the feature of channel c in the RGB branch, as shown in Equation (3). If $\gamma_{t,l,c}<\theta$ and $\gamma_{v,l,c}>\theta$, the feature of channel c in the RGB branch is used to replace the feature of channel c in the TIR branch, as shown in Equation (4).

(3) $${x^{\prime }}_{v,l,c}=\left\{\begin{array}{ll} \gamma_{v,l,c}\frac{x_{v,l,c}-\mu_{v,l,c}}{\sqrt{\sigma^{2}{}_{v,l,c}+\epsilon }}+\beta_{v,l,c}, & if\;\gamma_{v,l,c}>\theta ;\\ \gamma_{t,l,c}\frac{x_{t,l,c}-\mu_{t,l,c}}{\sqrt{\sigma^{2}{}_{t,l,c}+\epsilon }}+\beta_{t,l,c} & else\;if\;\gamma_{t,l,c}>\theta ; \end{array}\right.$$

(4) $$x{’}_{t,l,c}=\left\{\begin{array}{ll} \gamma_{t,l,c}\frac{x_{t,l,c}-\mu_{t,l,c}}{\sqrt{\sigma^{2}{}_{v,l,c}+\epsilon }}+\beta_{t,l,c}, & if\;\gamma_{t,l,c}>\theta ;\\ \gamma_{v,l,c}\frac{x_{v,l,c}-\mu_{v,l,c}}{\sqrt{\sigma^{2}{}_{v,l,c}+\epsilon }}+\beta_{v,l,c} & else\;if\;\gamma_{v,l,c}>\theta ; \end{array}\right.$$

**Figure 4:**
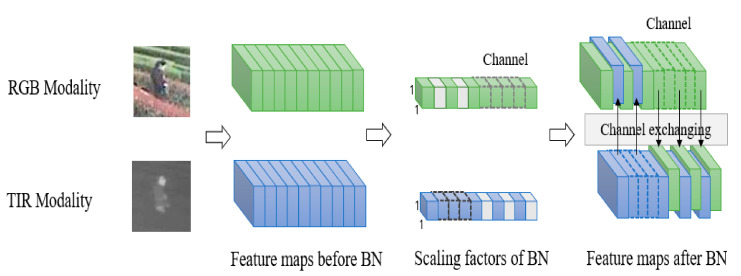
RGB and TIR dual-modal channel exchanging framework.

We apply Equations (3) and (4) to the RGB and TIR modalities, respectively, then put them into the nonlinear activation layer, and perform the convolution of the next layer. The gradient is separated from the replaced channel and propagates back through the new channel. In the implementation process, we apply the sparsity constraint of the scale factor to two disjoint regions of different modalities. The specific channel exchanging process is shown in Figure 4.

### 3.2. Network Architecture of the RGB-T Tracker Based on DiMP

Due to the excellent performance of DiMP [11] in terms of target discrimination ability and optimization speed, our proposed CEDiMP utilizes DiMP as the baseline tracker. DiMP is composed of two branches: the target classification branch is used to distinguish between the target and the background; the bounding box estimation branch is used to predict the accurate target bounding box. In the target classification branch, the depth features extracted from the training dataset and the testing dataset by the feature extractor F are transformed into specific classification features. Then, the feature map generated from the training dataset is input into the model predictor D (the predictor is composed of an initialization module and a loop optimization module). Effective weight initialization and fast gradient backpropagation make DiMP’s ability of discriminating targets and backgrounds significantly higher than ATOM [22]. The bounding box estimation branch of DiMP, like ATOM, is based on IoU-Net [57], utilizing the overlap-maximization strategy for accurate bounding box estimation.

**Figure 5:**
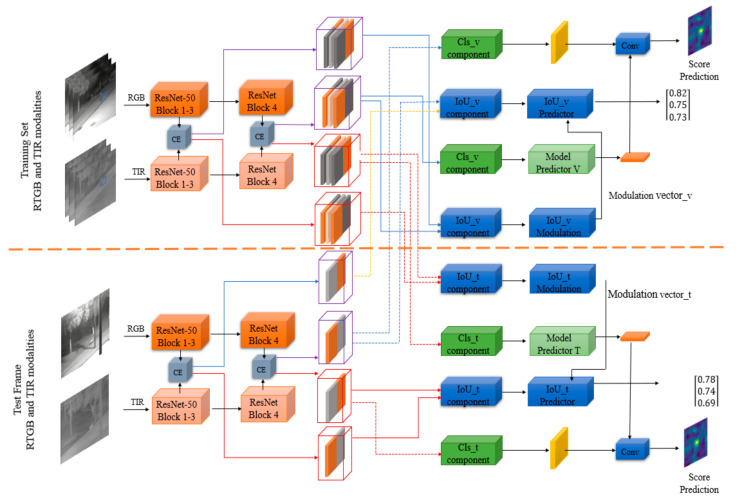
The framework of CEDiMP.

The training set and testing set of CEDiMP consists of RGB and TIR data. As shown in Figure 5, the images from each modality are input into the corresponding feature extraction network. The depth characteristics of RGB and TIR modalities are merged by channel exchanging. CE (channel exchanging) is a novel data fusion method. It is a parameter-free multi-modal data fusion method that can dynamically exchange channels between sub-networks of different modes. CE can achieve the feature representation model which has powerful multi-modal common features and single-modal unique features. In order to complete the precise location of the target, the depth features of RGB and TIR modalities after channel exchanging are adjusted to the features suitable for overlap rate maximization estimation through IoU_v component and IoU_t component. In order to achieve robust classification of targets and backgrounds, the depth features of RGB and TIR modalities after channel exchange are adjusted to features suitable for classification through Cls_v component and Cls_t component. In this way, it can provide a more expressive representation for IoU (intersection-over-union) prediction, and it can provide more distinctive features for the model predictor. The adjusted features are input to the IoU predictor (IoU_v predictor and IoU_t predictor) and the model predictor (model predictor V and model predictor T), respectively. Different from the existing RGB-T object tracking method, our proposed CEDiMP, respectively, supervises the training of the two branches, RGB and TIR. The classification and the bounding box estimation results of the RGB branch are shown in the upper part of Figure 5; the classification and the bounding box estimation results of the TIR branch are shown in the lower part of Figure 5.

### 3.3. The Training and Optimization of the Target Classification Sub-Network

**Model predictor optimization**. We define training set as $M_{train\_total}$, which contains two subsets, RGB subset $M_{train\_v}$ and TIR subset $M_{train\_t}$. $S_{train\_v}$ and $S_{train\_t}$ are generated by convolutional neural network feature extraction and channel exchanging operation on the input image, $S_{train\_v}={\left\{x_{v}^{\left(i\right)},c_{v}^{\left(i\right)}\right\}}_{i=1}^{n}$, $S_{train\_t}={\left\{x_{t}^{\left(i\right)},c_{t}^{\left(i\right)}\right\}}_{i=1}^{n}$. $x_{v}^{\left(i\right)}\in X$ and $x_{t}^{\left(i\right)}\in X$ are the classification features. The RGB feature extraction network and the TIR feature extraction network first complete the feature extraction process, respectively; then, the channel exchanging operation is performed; and finally, the extracted features are transformed into specific classification features $x_{v}^{\left(i\right)}\in X$, $x_{t}^{\left(i\right)}\in X$. $c_{v}^{\left(i\right)}\in {\mathbb{R}}^{2}$ and $c_{t}^{\left(i\right)}\in {\mathbb{R}}^{2}$ are the center coordinates of the RGB and TIR samples, respectively. In order for the model predictors of the RGB branch and the TIR branch to obtain the optimized filters $f_{v}$ and $f_{t}$, respectively, we initially utilize the original the least square loss, as shown in Equations (5) and (6).

(5) $$L\left(f_{v}\right)=\frac{1}{S_{train\_v}}\underset{\left(x_{v},c_{v}\right)\epsilon S_{train\_v}}{\sum }{\left|\left|r\left(x_{v}*f_{v},c_{v}\right)\right|\right|}^{2}+{\left|\left|\lambda f_{v}\right|\right|}^{2}$$

(6) $$L\left(f_{t}\right)=\frac{1}{S_{train\_t}}\underset{\left(x_{t},c_{t}\right)\epsilon S_{train\_t}}{\sum }{\left|\left|r\left(x_{t}*f_{t},c_{t}\right)\right|\right|}^{2}+{\left|\left|\lambda f_{t}\right|\right|}^{2}$$

where ∗ represents the convolution operation, and *λ* is the regularization factor. The function r in Equation (5) is used to calculate the residual difference between the predicted target confidence score in the RGB branch and the true target center coordinates; the function r in Equation (6) is used to calculate the residual difference between predicted target confidence score in the TIR branch and the true target center coordinates. During the training process, we have found that simply taking the difference would force the model to regress to the corrected confidence value for all negative samples. This makes learning focus on negative data samples instead of obtaining the best discrimination ability. In the object tracking task, the numbers of positive and negative samples are unbalanced. In order to solve the imbalance problem of the numbers of positive and negative samples, we use hinge-like loss (l) in the calculation of r. When solving the optimal solution of $f_{v}$ and $f_{t}$, we do not use the common stochastic gradient descent method, instead utilizing the steepest descent method referenced from DiMP. Adopting the steepest descent method can obtain powerful filters $f_{v}$ and $f_{t}$ after several iterations.

**Offline training.** Different from the existing RGB-T object tracking framework, in order to make the target discrimination branch more robust, both the RGB and TIR branches of CEDiMP utilize multiple frames in the video sequence to form the training set and the testing set. The RGB branch randomly selects a subsequence of length T from the RGB sequence. The former part of the subsequence forms the training set $M_{train\_v}$, and the later part forms the testing set $M_{test\_v}$. The training set $M_{train\_t}$ and testing set $M_{test\_t}$ of the TIR branch are generated with the same strategy as the RGB branch. After the offline training starts, the RGB branch and the TIR branch perform the same operations. The paired $\left(M_{train_{v}},M_{test\_v}\right)$ would generate corresponding $\left(S_{train_{v}},S_{test_{v}}\right)$ after feature extraction and channel exchanging; similarly, the paired $\left(M_{train_{t}},M_{test_{t}}\right)$ generates corresponding $\left(S_{train_{t}},S_{test_{t}}\right)$ after feature extraction and channel exchanging. $S_{train\_v}$ and $S_{train\_t}$ provide training data for the model predictor in order to obtain $f_{v}$ and $f_{t}$ with strong discrimination ability. Testing samples in the RGB and TIR modalities $S_{test\_v}$ and $S_{test\_t}$ are used to evaluate the filters $f_{v}$ and $f_{t}$, and the final target classification loss is calculated by the mean square error of all testing samples. Equations (7) and (8) show the classification loss used in the offline training of the RGB and TIR modalities, respectively.

(7) $$L_{cls\_v}=\frac{1}{N_{iter}}\overset{N_{iter}}{\underset{j=0}{\sum }}\underset{\left(\left(x_{v},c_{v}\right)\right)\epsilon S_{test\_v}}{\sum }{\left|\left|l\left(x_{v}*f_{v}{}^{\left(j\right)},z_{c\_v}\right)\right|\right|}^{2}$$

(8) $$L_{cls\_t}=\frac{1}{N_{iter}}\overset{N_{iter}}{\underset{j=0}{\sum }}\underset{\left(\left(x_{t},c_{t}\right)\right)\epsilon S_{test\_t}}{\sum }{\left|\left|l\left(x_{t}*f_{t}{}^{\left(j\right)},z_{c\_t}\right)\right|\right|}^{2}$$

$N_{iter}$ is the number of planned optimization iterations, l() is hinge-like loss, the regression label $z_{c\_v}$ represents the Gaussian function centered on the RGB target c, and the regression label $z_{c\_t}$ represents the Gaussian function centered on the TIR target c. Note that we not only evaluate the final target model f, but also evaluate the average loss of estimated $f^{\left(j\right)}$ obtained by the optimizer in each iteration j. Introducing intermediate supervision into the target prediction module is beneficial for the convergence of the training process. In addition, our goal is not to train a specific number of recursions, but to freely set the number of required recursions.

### 3.4. Bounding Box Estimation Branch

We take advantage of the overlap maximization strategy to perform accurate bounding box estimation. Given the appearance of the reference object, the bounding box estimation branch is trained to predict the IoU overlap between the target and previous set of candidate boxes on the testing image. The calculated vector is used to modulate the features in testing images, and then it is utilized for IoU prediction. Different from the existing RGB-T object tracking method, we simultaneously supervise the RGB and TIR modalities during network training and maximize the IoU between the predicted bounding box and the true value of each modality, respectively. The calculation methods of IoU between the single predicted bounding box and the true value of the RGB and the TIR modalities are shown in Equation (9) and Equation (10), respectively:

(9) $$IoU\left(B_{v}\right)=g\left(w\left(x_{v0},B_{v0}\right)\cdot z\left(x_{v},B_{v}\right)\right)$$

(10) $$IoU\left(B_{t}\right)=g\left(w\left(x_{t0},B_{t0}\right)\cdot z\left(x_{t},B_{t}\right)\right)$$

$x_{v0},B_{v0}$ come from the first frame of $M_{train\_v}$, and $x_{v},B_{v}$ are obtained by randomly sampling an image frame in $M_{test\_v}$. $x_{t0},B_{t0}$. come from the first frame of $M_{train\_t}$, and $x_{t},B_{t}$ are obtained by randomly sampling an image frame in $M_{test\_t}$. w is the modulation vector, z is the feature representation of the single image frame processed by the PrPool [57] layer in the testing set, and g is the IoU predictor with three fully connected layers. The target information is integrated into the IoU prediction by computing a modulation vector from the reference appearance of the target. The bounding box estimation loss of the RGB and TIR modalities, $L_{bb\_v}$ and $L_{bb\_t}$, are the squared errors between the predicted IoU overlap and the true value of all samples in $M_{test\_v}$ and $M_{test\_t}$, respectively.

### 3.5. Final Loss Function

In order to achieve a balance between accuracy and robustness of the object tracking task in both RGB and TIR modalities, we perform supervision in both RGB and TIR modalities during the offline training process, instead of compromising between the two modalities as in the existing RGB-T object tracking methods. The total loss of the RGB modality is the value of $L_{tot\_v}$, as shown in Equation (11). The total loss of the TIR modality is the value of $L_{tot\_t}$, as shown in Equation (12). $\varphi_{v}$ and $\varphi_{t}$ are hyperparameters set to increase the impact of the classification loss on the total loss. The loss function of the CEDiMP framework is the sum of the loss in the RGB and TIR modalities, as shown in Equation (13).

(11) $$L_{tot\_v}=\varphi_{v}L_{bb\_v}+L_{cls\_v}$$

(12) $$L_{tot\_t}=\varphi_{t}L_{bb\_t}+L_{cls\_t}$$

(13) $$L_{tot}=L_{tot\_v}+L_{tot\_t}$$

### 3.6. LaSOT-RGBT

From Table 1, we can see that the performance of the current RGB-T object tracking frameworks on unknown datasets decreases significantly, which shows that the RGB-T object tracking frameworks generally have poor generalization ability. In addition, we have found that, although mfDiMP only ranked third in EAO on the public dataset in the VOT-RGBT2019 challenge, the EAO of mfDiMP on the sequestered dataset ranked first. After analysis, it is found that the top five trackers all use depth features. However, mfDiMP is the only tracker that has performed the neural network training on the large-scale synthetic dataset GOT10K-RGBT. This demonstrates that only training on the small-scale RGB-T datasets RGBT234 and GTOT cannot make the tracker obtain strong generalization capabilities. When the testing videos contain the conditions that target reappears after disappearing from the field of view, the target state transits, and so on, all the RGB-T trackers in Table 1 would fail. This indicates that the current methods cannot cope with the typical challenges of long-term object tracking tasks. In order to improve the generalization ability and long-term object tracking ability of the RGB-T tracker, we first generate the dataset of the TIR modality LaSOT-TIR with the trained image translation model based on the long-term object tracking dataset of RGB modality LaSOT. Through the above steps, we have obtained the synthetic dataset LaSOT-RGBT, which can be used for RGB-T long-term tracking. Compared with mfDiMP, our proposed CEDiMP framework is also trained on LaSOT-RGBT. With extra training on the large long-term object tracking dataset, CEDiMP not only has the ability to deal with challenges such as the reappearance of the target after disappearing from the field of view and the transition of target state, but also further improves the generalization ability.

## 4. Experiments

In order to verify the effectiveness of our proposed method, we have conducted many representative experiments. All experiments have been performed on a PC equipped with NVIDIA TITAN X GPU and i7-9600K CPU. We have implemented CEDiMP on PyTorch.

### 4.1. Implementation Details

#### 4.1.1. Backbone Network

The backbone network of the CEDiMP tracking framework is ResNet50 [58], but only the first 4 blocks are used. In order to make the feature representation model of the tracker obtain the powerful representation capabilities of multi-modal common features and single-modal unique features, we perform channel exchanging operations in the backbone network of the RGB and TIR modalities when completing feature extraction tasks. The output features of Block3 and Block4 are used to estimate the bounding box, but only the output features of Block4 are utilized to classify the target and background.

#### 4.1.2. Offline Training

In the offline training process, GOT10K-RGBT and LaSOT-RGBT are used for training. GOT10K-RGBT and LaSOT-RGBT contain 8335 and 1120 pairs of video sequences aligned with visible light and thermal infrared, respectively. The final loss function of offline training is shown in formula 13. The RGB branch inputs three image pairs each time, i.e., $M_{train_{v}}=3,M_{test_{v}}=3$; the TIR branch performs the same operation, i.e., $M_{train_{t}}=3,M_{test_{t}}=3$. In order to speed up the convergence of the neural network during the training process, we have utilized the DiMP pre-trained model. The parameters of the RGB branch and the TIR branch are fine-tuned, respectively, during training. Since the pre-trained model is generated based on the RGB modality, the learning rate of the TIR branch of CEDiMP is greater than that of the RGB branch (in this paper, lr_t_ = 10lr_v_, where lr_v_ is the learning rate of the RGB branch, and lr_t_ is the learning rate of the TIR branch), so that the two modalities can learn the optimal results at the same time. The initial learning rate of the RGB branch is lr_v_ = 10^−3^, and the initial learning rate of the TIR branch is lr_t_ = 10^−2^. The entire training process contains 50 epochs, and the learning rate drops by 0.1 every 10 epochs. To increase the speed of CEDiMP, we have to sacrifice some accuracy, so we set $N_{iter}$ to be 4.

#### 4.1.3. Online Tracking

During online tracking, the RGB and TIR branches are given the first frame with annotations, respectively. We use the data augmentation strategy to construct two initial sets $S_{train\_v}$ and $S_{train\_t}$ containing 15 samples, respectively. $S_{train\_v}$ and $S_{train\_t}$ are input into the model predictor V and the model predictor T, respectively, and the purpose is to obtain filters with strong discrimination ability $f_{v}$ and $f_{t}$ after several iterations. For the first frame of the RGB and TIR modalities, we all utilize the deepest descending recursion four times after initialization of the module. The sizes of $S_{train\_v}$ and $S_{train\_t}$ are always 30, but the samples inside are constantly updated. In the tracking process, the samples whose confidence meet the requirements would be added to $S_{train\_v}$ and $S_{train\_t}$, and the original samples in $S_{train\_v}$ and $S_{train\_t}$ would also be abandoned at the same time. During the tracking process, we make sure that $f_{v}$ and $f_{t}$ have a strong discrimination ability by performing recursions of the optimization program twice every 20 frames or performing one recursion when the interference peak is detected. The bounding box estimation branches of the two modalities perform the same operation as ATOM.

### 4.2. Comparison to State-of-the-Art Trackers

In order to validate the effectiveness of the proposed RGB-T object tracking framework based on channel exchanging data fusion, we make a detailed comparison with other excellent trackers on two RGB-T object tracking benchmark datasets. GTOT [3] has 15.8K frames, including 50 RGB-T videos aligned spatially and temporally and seven labeled attributes. RGBT234 [2] has 234K frames, 234 aligned RGB-T videos, and 12 labeled attributes. When compared with other methods, two common metrics, precision (PR) and success rate (SR), are utilized to evaluate the performance of the tracker. Since CEDiMP can separately supervise the RGB branch and the TIR branch, two precision results, including PR_v_ (the precision of the RGB branch) and PR_t_ (the precision of the TIR branch), and two success rate results, including SR_v_ (success rate of RGB branch) and SR_t_ (success rate of TIR branch), would be generated. For a fair comparison with other methods, we set PR = (PR_v_ + PR_t_)/2 and SR = (SR_v_ + SR_t_)/2.

#### 4.2.1. Evaluation on GTOT Dataset

**Figure 6:**
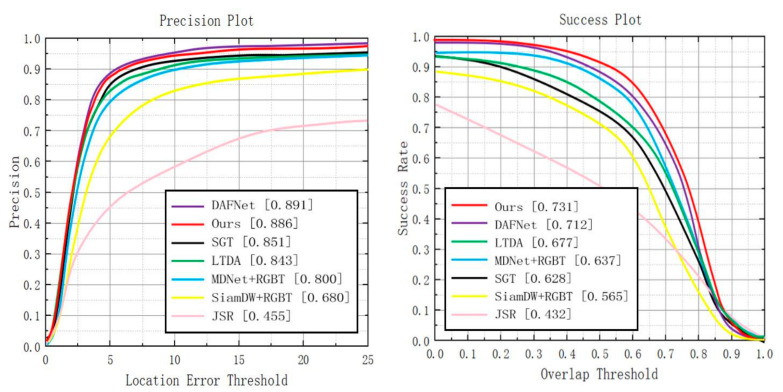
Comparison results with the current state-of-the-art methods on GTOT.

We have compared CEDiMP with six state-of-the-art trackers on GTOT. Since the targets in the GTOT dataset are always small, we set the threshold of PR to be five pixels. It can be seen from Figure 6 that the proposed CEDiMP achieves the runner-up performance in PR, only lower than the first ranked DAFNet [59] by 0.56%, and higher than the third place SGT [45] by 4.11%. However, CEDiMP achieves the best performance in SR, which is 2.67% higher than the second place DAFNet and 7.98% higher than the third place LTDA [60].

#### 4.2.2. Evaluation on REGT234 Dataset

**Figure 7:**
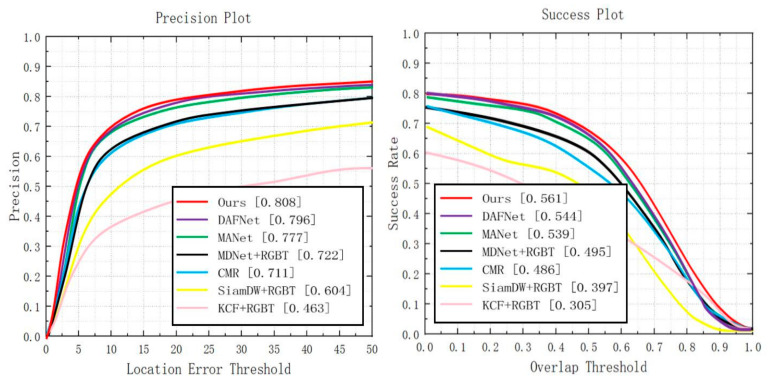
Comparison results with the current state-of-the-art methods on GRBT234.

**Overall performance.** When performing comparative experiments on RGBT234, we set the threshold of PR to be 20 pixels. After compared with other six state-of-the-art trackers, it is found that the proposed CEDiMP achieves the best performance in both PR and SR. The PR of our model is 1.51% higher than the second place DAFNet and 3.99% higher than the third place MANet [7]. The SR of our model is 3.13% higher than the second place DAFNet and 4.08% higher than the third place MANet. The details are shown in Figure 7.

**Figure 8:**
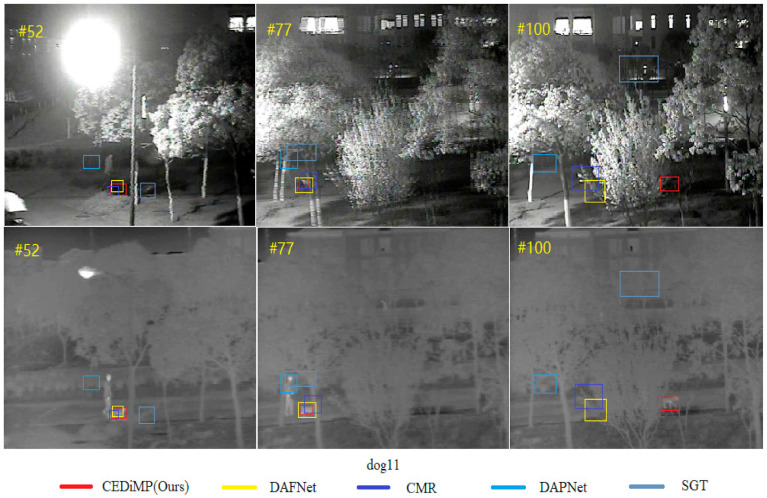
Qualitative comparison results on dog11 image sequences in RGBT234.

**Figure 9:**
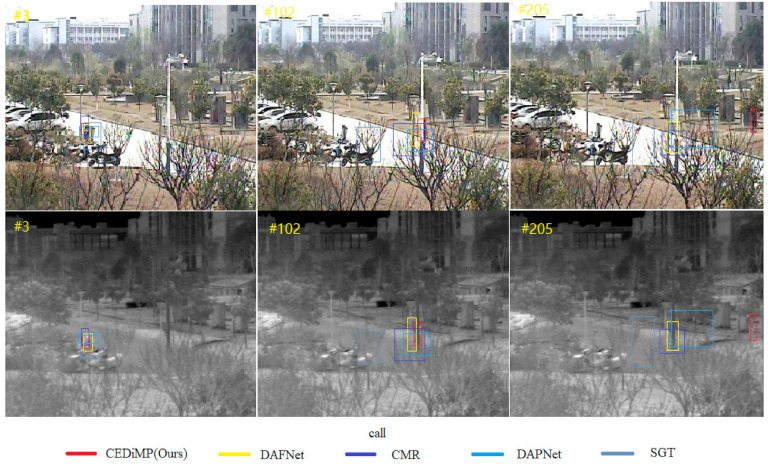
Qualitative comparison results on call image sequences in RGBT234.

**Attribute-based performance.** Excitingly, the creators of RGBT234 have annotated attributes for each video sequence in order to complete attribute-sensitive performance analysis. RGBT234 annotates the sequence with 12 attributes, which represent 12 challenging aspects in visual tracking. In order to evaluate the specific performance of CEDiMP in 12 challenges of the object tracking task, we compare the proposed model with the most advanced RGB-T trackers. The specific results are shown in Table 3. The comparison results indicate that the overall performance of CEDiMP is the optimal, especially solving challenges such as occlusion, low illumination, image blur, and quick movement. In order to demonstrate the excellent performance of CEDiMP on these challenges more intuitively, we have selected the dog11 and call video sequences in RGBT234 to perform a qualitative comparison, as shown in Figure 8 and Figure 9.

Although the proposed model achieves better performance on GTOT and RGBT234, we hope that the performance of CEDiMP can degrades less than other methods on datasets similar to the sequestered dataset in the VOT-RGBT2019 challenge, because sequestered datasets can evaluate the performance of an RGB-T tracker more objectively in an open environment. The numbers of video sequences in GTOT and RGBT234 are not enough, the scene type is much unitary, and the video sequences are generally short. Such benchmark dataset is not convincing enough to accurately measure the real performance of the tracker in an open environment.

**Figure 10:**
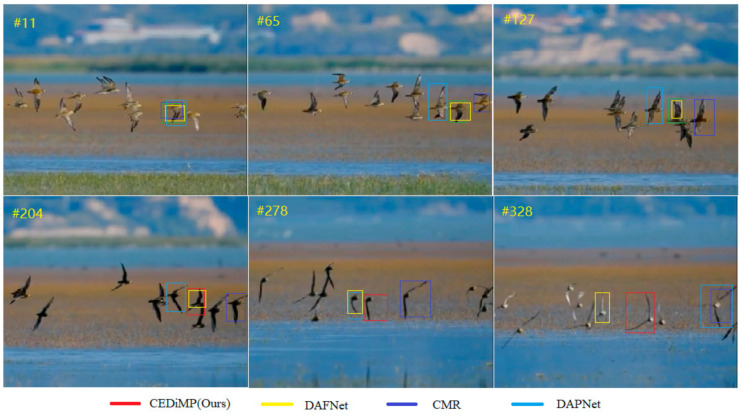
Qualitative comparison results of single RGB modality.

Since we cannot obtain the sequestered dataset of the VOT-RGBT2019 challenge, we have downloaded a video of RGB modality with serious interference of similar objects from the Internet. The comparison results on this video are shown in Figure 10. We can see that CMR and DAPNet have drifted to the interference object at the 65th frame, while CEDiMP and DAFNet can track the target object correctly. However, since the 278th frame, all the trackers have drifted to the interference object except for the proposed CEDiMP.

**Figure 11:**
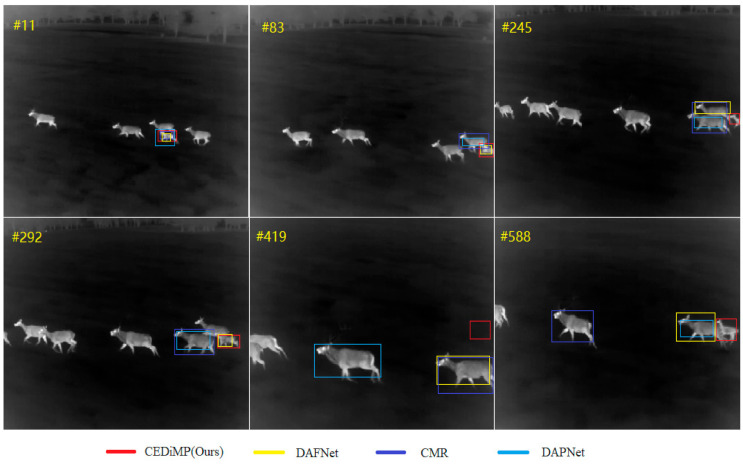
Qualitative comparison results of single TIR modality.

In order to evaluate the performance of CEDiMP on TIR single-modal sequestered video, we have selected a video sequence containing the challenge of the reappearance of the target object after leaving the field of view from the recent public TIR single object tracking benchmark dataset LSOTB-TIR [61] in order to perform a comparison testing. The tracking object, the deer, left the field of view twice before the 588th. As shown in Figure 11, although CEDiMP, DAFNet, CMR, and DAPNet can still track the object at the 11th frame, CMR and DAPNet drift to other deer at the 83rd frame. As the deer gradually leaves the field of view, DAFNet has drifted to other deer at the 245th frame. Although DAFNet has captured the target again at the 292nd frame, we can infer from the subsequent tracking results of the 419th frame and the 588th frame that this would be an accidental result.

**Figure 12:**
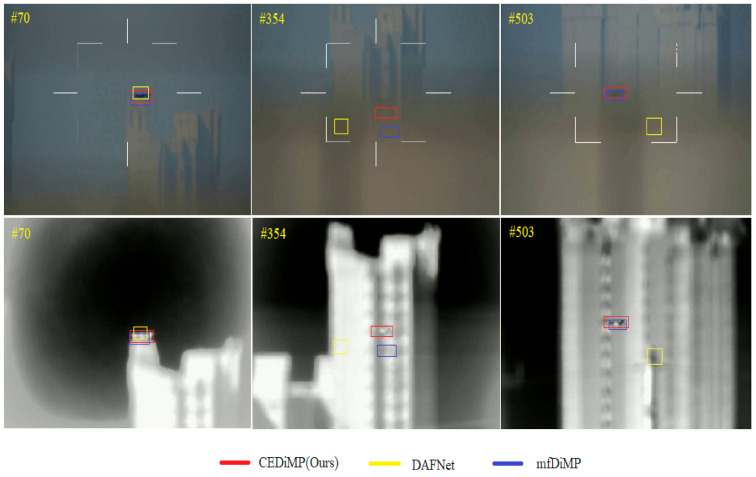
Qualitative comparison results of RGB and TIR modalities.

To verify the performance of CEDiMP on RGB and TIR dual-mode sequestered video, we have selected a difficult video from the testing set in the first Anti-UAV Challenge to complete the comparison experiment. In this video, the camera teleports for many times, the resolution of images is low, and the target object often disappears in single-modal images. In order to validate whether the feature fusion based on channel exchanging is better than that based on aggregation, we have deliberately completed the qualitative comparison with mfDiMP. It can be seen from Figure 12 that DAFNet achieves excellent performance on GTOT and RGBT234 but performs the worst in this performance comparison. The target is not tracked correctly at the 354th frame and the 503rd frame, which indicates that the generalization performance of DAFNet is poor. Both mfDiMP and CEDiMP have tracked the target correctly at the 70th frame and the 503rd frame, but mfDiMP has drifted to the background at the 354th frame.

### 4.3. Ablation Study

To evaluate the impact of multi-modal data fusion input on object tracking, the effectiveness of the proposed CE module, and the benefit on the improvement of the tracker’s performance with extra training on LaSOT-RGBT, we have conducted the ablation study on the RGBT234 benchmark dataset, which is widely used when evaluating the performance of the RGB-T tracker.

**Figure 13:**
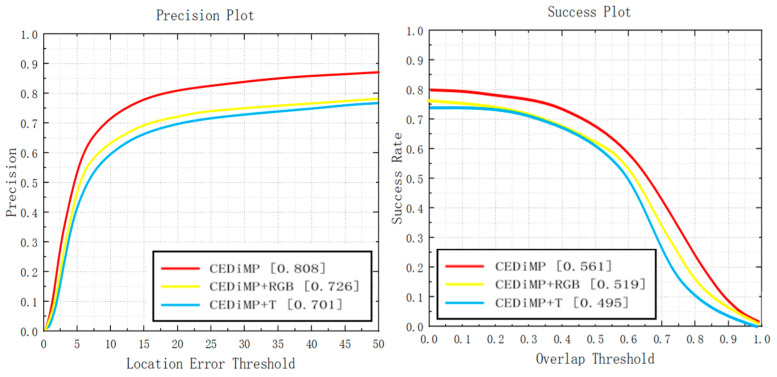
Single/dual-modal experiments results.

**Single/dual-modal data.** Although RGB cameras can obtain images of high resolution, gain rich image texture and color features, they cannot perform well in specific environments such as low illumination, strong light, rain, and haze. TIR cameras can obtain images with higher quality under low illumination, strong light, rain, and haze, but TIR images are temperature-sensitive, have low resolution, and easily lose information, such as colors, target edges, and geometric textures. There have been many challenges in the object tracking task in the all-weather, open environment. Thus, we guess that it is difficult for the tracker to achieve the best performance only with the single modal input of RGB or TIR data. The experiment results in Figure 13 demonstrate our ideas. CEDiMP with dual-modal input has achieved better performance than any tracker with single-modal input in both PR and SR (CEDiMP+RGB represents that only RGB images are input, and CEDiMP+T represents that only TIR images are input).

**Figure 14:**
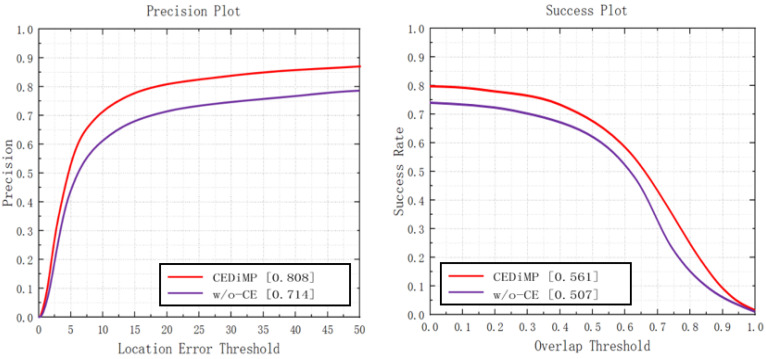
Comparison results w/o-CE.

**Figure 15:**
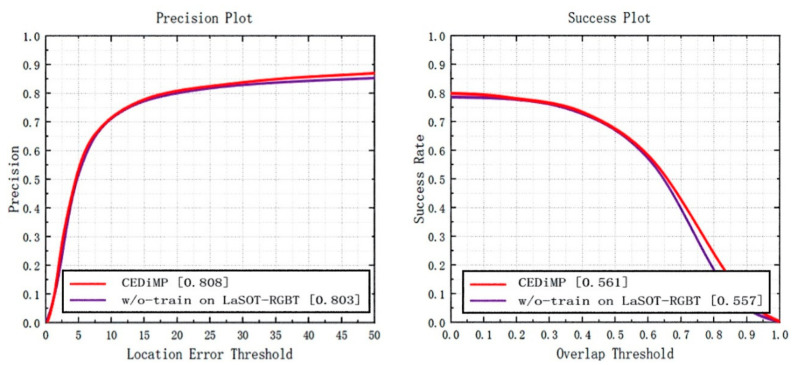
Comparison results w/o-training on LaSOT-RGBT.

**Prune experiments.** In order to verify the effectiveness of the proposed CE module in RGB-T object tracking, we have removed the CE module in CEDiMP and have performed the comparison experiments. It can be seen from Figure 14 that the CE module has the significant impact on improving the performance of the tracker. Without the CE module, the PR of the model would reduce by 13.17%, and the SR of the model would reduce by 10.65%. From Figure 15, we can find that the difference between the model performance in PR and SR on RGBT234 is not obvious regardless of whether CEDiMP is trained on LaSOT-RGBT additionally.

## 5. Discussion

The results of GTOT and RGBT234 demonstrate that the proposed CEDiMP achieves the best performance, but the advantages are not obvious. However, the qualitative comparison results on sequestered videos indicate that CEDiMP has obvious advantages. The effect of the baseline tracker of the RGB-T tracker is of vital importance and cannot be ignored, as shown in Figure 10. If the discrimination ability of the baseline tracker is not strong enough, the tracker will easily drift to interference objects that have high similarity with the target, no matter how the data are fused. CEDiMP utilizes DiMP as the baseline tracker, and the most prominent characteristic of DiMP is that it can ensure the optimal discriminative ability of the discriminator all the time with the efficient online learning method. This is the main reason for the obvious advantages of CEDiMP in Figure 10. The video sequences in Figure 11 contain the typical long-term object tracking challenge, and the target leaves the field of view twice before the 588th frame. From the performance of each tracker in Figure 11, we can find that, except for the baseline of the tracker, the training of the tracker on the long-term object tracking dataset LaSOT-RGBT is helpful to solve the challenges in the long-term object tracking task. However, if the evaluation data samples only contain the video sequences with a short frame length and do not contain the video sequences with the long-term object tracking challenges, training on LaSOT-RGBT would not significantly improve the performance. Figure 15 shows the results of the ablation experiments on the RGBT234 benchmark dataset. The above reasons are the reasons that the training on LaSOT-RGBT cannot significantly improve the performance of the tracker.

The quality of RGB and TIR data fusion directly determines the performance of the RGB-T tracker. Figure 8, Figure 9 and Figure 12 can demonstrate the advantages of fusing RGB and TIR data by channel exchanging. Channel exchanging is a multi-modal data fusion method with no parameter that can dynamically exchange channels between different modes of sub-networks, which makes our feature representation model have powerful representation ability in multi-modal common features and single-modal unique features. As shown in Figure 12, we directly compare CEDiMP and mfDiMP with the same baseline tracker. The main reason that mfDiMP drifts to the background at the 354th frame is that the data fusion method is not efficient enough. mfDiMP directly concatenates the depth features of the RGB mode and the TIR mode and utilizes 1 × 1 convolution to reduce the dimensionality. Then, the fused features are input into the IoU predictor and model predictor. The hyperparameter-based feature aggregation method would reduce the model’s representation ability in the unique features of the original modality. This feature fusion method limits the improvement of its performance.

## 6. Conclusions

In this paper, we propose an RGB-T tracker CEDiMP based on bimodal data fusion by channel exchanging. Our method completes dynamic channel exchanging between sub-networks of different modes without adding any parameters during feature fusion. Since we use DiMP as the baseline tracker, CEDiMP is very powerful in distinguishing targets and backgrounds. Considering that most RGB-T trackers have poor generalization abilities currently, we firstly utilize the trained image translation model to generate TIR modality dataset LaSOT-TIR based on the RGB modality long-term object tracking dataset LaSOT. Then, we obtain the synthetic dataset LaSOT-RGBT, which can be used for RGB-T long-term tracking. The extra training of CEDiMP on LaSOT-RGBT improves the ability of solving the typical challenges of long-term object tracking and significantly improves the generalization ability of the model. Our tracker not only achieves the best performance on GTOT and RGBT234, but also significantly outperforms other trackers in some qualitative tests of sequestered videos.

## Figures and Tables

**Table 1:** The Top 5 EAO in the VOT-RGBT2019 Challenge.

RGB-T Tracker Name	EAO on the Public Dataset VOT-RGBT2019	EAO on the Sequestered Dataset	Final Ranking in the VOT-RGBT2019 Challenge
mfDiMP [5]	0.3879	0.2347	1
siamDW_T [6]	0.3925	0.2143	2
MANet [7]	0.3436	0.2041	3
JMMAC [8]	0.4826	0.2037	4
FSRPN [9]	0.3553	0.1873	5

**Table 2:** Examples of recent published research on RGB-T trackers.

References	Years	Journal/Conference	Category
[38]	2007	IEEE Conference on Computer Vision and Pattern Recognition	Traditional method
[39]	2011	IEEE International Conference on Information Fusion	Traditional method
[40]	2018	Conference on Image and Graphics Technologies and Applications	Sparse representation (SR)-based
[41]	2017	IEEE Transactions on Systems, Man, and Cybernetics: Systems	Sparse representation (SR)-based
[42]	2018	In Proceedings of the European Conference on Computer Vision	Sparse representation (SR)-based
[43]	2011	IEEE International Conference on Information Fusion	Sparse representation (SR)-based
[44]	2012	Science China Information Sciences	Sparse representation (SR)-based
[45]	2017	In Proceedings of the ACM international conference on Multimedia	Graph-based
[46]	2019	IEEE Transactions on Circuits and Systems for Video Technology	Graph-based
[47]	2018	Signal Processing: Image Communication	Graph-based
[48]	2019	Neuro computing	Correlation Filter (CF)-based
[49]	2018	In Pattern Recognition and Computer Vision	Correlation Filter (CF)-based
[50]	2019	Infrared Physics & Technology	Correlation Filter (CF)-based
[51]	2019	Mathematical Problems in Engineering	Correlation Filter (CF)-based
[52]	2020	IEEE Conference on Computer Vision and Pattern Recognition	Deep Learning (DL)-based
[53]	2020	European Conference on Computer Vision	Deep Learning (DL)-based
[54]	2020	Sensors	Deep Learning (DL)-based
[55]	2019	IEEE International Conference on Image Processing	Deep Learning (DL)-based
[56]	2019	ACM international conference on Multimedia	Deep Learning (DL)-based

**Table 3:** PR/SR scores (%) based on attributes. The best, second, and third performances are shown in red, green, and blue, respectively.

	CMR [42]	DAPNet [61]	SGT [45]	DAFNet [59]	CEDiMP (Ours)
NO	89.5/61.6	90.0/64.4	87.7/55.5	90.0/63.6	88.1/65.9
PO	77.7/53.5	82.1/57.4	77.9/51.3	85.9/58.8	87.1/60.5
HO	56.3/37.7	66.0/45.7	59.2/39.4	68.6/45.9	69.8/46.1
LI	74.2/49.8	77.5/53.0	70.5/46.2	81.2/54.2	82.5/55.1
LR	68.7/42.0	75.0/51.0	75.1/47.6	81.8/53.8	78.8/53.2
TC	67.5/44.1	76.8/54.3	76.0/47.0	81.1/58.3	81.4/55.0
DEF	66.7/47.2	71.7/51.8	68.5/47.4	74.1/51.5	73.1/52.9
FM	61.3/38.2	67.0/44.3	67.7/40.2	74.0/46.5	75.4/48.2
SV	71.0/49.3	78.0/54.2	69.2/43.4	79.1/54.4	78.2/55.8
MB	60.0/42.7	65.3/46.7	64.7/43.6	70.8/50.0	72.1/50.6
CM	62.9/44.7	66.8/47.4	66.7/45.2	72.3/50.6	72.5/52.1
BC	63.1/39.7	71.7/48.4	65.8/41.8	79.1/49.3	80.1/51.2
ALL	71.1/48.6	76.6/53.7	72.0/47.2	79.6/54.4	80.8/56.1
